# Ontogeny drives shifts in skin bacterial communities in facultatively paedomorphic salamanders

**DOI:** 10.1099/mic.0.001399

**Published:** 2023-10-10

**Authors:** Arik M. Hartmann, Sarah E. McGrath-Blaser, Zuania Colón-Piñeiro, Ana V. Longo

**Affiliations:** ^1^​ Department of Biology, University of Florida, Gainesville, Florida, USA

**Keywords:** amphibians, chytrid, development, microbiome, paedomorphosis

## Abstract

Microbiomes are major determinants of host growth, development and survival. In amphibians, host-associated bacteria in the skin can inhibit pathogen infection, but many processes can influence the structure and composition of the community. Here we quantified the shifts in skin-associated bacteria across developmental stages in the striped newt (*Notophthalmus perstriatus*), a threatened salamander species with a complex life history and vulnerable to infection by the amphibian chytrid fungus *Batrachochytrium dendrobatidis* and ranavirus. Our analyses show that pre-metamorphic larval and paedomorphic stages share similar bacterial compositions, and that the changes in the microbiome coincided with physiological restructuring during metamorphosis. Newts undergoing metamorphosis exhibited microbiome compositions that were intermediate between paedomorphic and post-metamorphic stages, further supporting the idea that metamorphosis is a major driver of host-associated microbes in amphibians. We did not find support for infection-related disruption of the microbiome, though infection replicates were small for each respective life stage.

## Data Summary

Raw sequence data files (Illumina reads) for all samples have been deposited in the NCBI SRA under BioProject PRJNA971630. The list of Biosample and SRA accession numbers for these samples is presented in Table S1. The authors confirm all supporting data and protocols have been provided within the article or through supplementary data files. Commands and scripts will be made available on the GitHub repository: https://github.com/amphibiarik/ontogeny_micro_shift.

**Impact Statement**

Our study examines shifts in skin bacterial communities across ontogeny in a threatened salamander species with complex and plastic developmental routes. Most studies in amphibians have focused on describing how microbial diversity changes before and after metamorphosis. However, we know comparatively little about how alternative developmental routes and pathogen infection shape the composition of host-associated microbes. In this study, we profiled skin bacterial communities of different life stages including paedomorphic salamanders, which retain larval features, become sexually mature and remain in aquatic habitats. We found that the onset of metamorphosis was the primary driver of differences in bacterial composition of newts. Aquatic life stages exhibit different patterns of susceptibility to infection by pathogens such as the amphibian chytrid fungi and ranaviruses, which have been involved in global amphibian declines. Although we did not find support for pathogen influence on bacterial composition, pathogen-induced bacterial disruption is well established in the literature. Our findings suggest that any impacts of infection could be masked by stronger structuring pressures such as metamorphosis.

## Introduction

The vertebrate microbiome is instrumental in maintaining host health and forms an important component of innate immune defence for many taxa [1–5]. In amphibians, certain host-associated microbes in the skin can inhibit the growth of pathogens [5–8], but their effectiveness in preventing disease relies on specific patterns of composition and abundance [9, 10]. Many studies over the past 20 years have established the various host and environmental factors that shape the composition of the amphibian microbiome. Ontogenetic shifts [11] can cause predictable transitions in amphibian skin and gut microbial communities, particularly before and after metamorphosis [12–16]. The process of metamorphosis in amphibians accompanies physiological and structural reorganization in most organ systems, including the skin, while concurrently inducing immunosuppression [17, 18]. Along with the physiological and anatomical restructuring, many amphibians undergo behavioural or habitat changes during metamorphosis, from larval stages restricted to aquatic environments, to adults that are terrestrial or semi-aquatic. These shifts in habitat occupancy through development influence the composition of the skin microbiota as environmental species pools are a major source of microbial recruitment for hosts [19].

In some amphibian species, ontogeny does not always reflect changes in host habitat, as many salamanders have facultative or obligate paedomorphic forms [20–22]. These differences in physiological and morphological traits probably alter microbial recruitment and colonization compared to metamorphosing forms. Paedomorphic forms retain larval-type skin and physiology and are restricted to aquatic environments [23–25] which constrains the influence of the microbiome composition compared to terrestrial and semi-aquatic forms. It remains unclear if microbiome composition differs between larval and paedomorphic stages, and when the shift in composition begins relative to metamorphosis. In addition, microbial colonization can include putative pathogens that use the developmental window of host immunosuppression as a way to establish in tissues [26]. Thus, it is important to integrate temporal and ontogenetic processes in studies of microbial composition and host immunity, especially in species with plastic developmental routes.

Amphibians experience periods of increased infection prevalence of pathogens, such as *Batrachochytrium dendrobatidis* (*Bd*) and ranaviruses (Rv), in specific life stages [27–34]. These periods may indicate ontogenetic drivers of host immune function, including shifts in the abundance of beneficial skin microbes during development [35, 36], which can alter susceptibility to pathogen infection [37, 38]. Most of the work examining amphibian microbiomes and immunity has focused on the susceptibility of a single life stage to *Bd* [4, 19], while studies incorporating a range of development stages and other pathogens are uncommon [16, 36, 39]. Determining the contributions of processes that influence microbial assembly and disruption is essential to understand how host–microbe interactions shape variable patterns of disease through seasonal and developmental shifts.

Here, we analysed changes in the skin bacterial community of the striped newt (*Notophthalmus perstriatus*), a threatened salamander species that has experienced range-wide population declines [40], potentially from emerging diseases [41]. *N. perstriatus* is a facultatively paedomorphic species with complex life histories that include gilled larval and paedomorphic stages, and post-metamorphic juveniles (efts) and adults [42]. While efts and adults are primarily terrestrial, adults will make annual migrations to breeding ponds where they overlap spatiotemporally with paedomorphic and larval stages. The complex development of *N. perstriatus* and the habitat overlap between the different life stages allow us to examine how the skin bacterial community shifts during life stage transitions and identify host-associated drivers of skin bacterial assembly. Additionally, because *N. perstriatus* populations are exposed to both *Bd* and Rv [41], we evaluated if skin microbial diversity and composition were influenced by pathogen infection, which could either homogenize communities or potentially increase diversity due to secondary infections. We also hypothesized that *N. perstriatus* skin microbial composition would change across life stages, where the most significant changes would be observed between pre- and post-metamorphic stages.

## Methods

### Sample collection

Wild striped newts were sampled from Putnam Country, Florida (USA), between February 2020 and May 2021 (Table S1, available in the online version of this article). Newts were captured by dip netting in aquatic vegetation and categorized into four distinct life stages (i.e. larva, paedomorph, metamorphosing and adult) based on morphological traits such as the presence of gills, skin granularity and secondary sexual characteristics. To examine microbial communities and assess pathogen infection dynamics, we collected skin swabs from individual amphibians using rayon-tipped swabs (Medical Wire and Equipment MW002NF). Each animal was swabbed for 10 strokes across the ventral surfaces of the body and hindlimbs, and five strokes each on the ventral and lateral surfaces of the tail to standardize sample collection. Animals were not rinsed before swabbing as these animals were already in water, and our goal was to quantify entire bacterial communities, not only resident taxa. We used new gloves between sampling each animal to prevent cross-contamination of samples. The tips of swabs were stored individually in sterile 2.0 ml screwcap tubes and transported to the University of Florida and stored at −20 °C until DNA extraction.

### DNA extraction and pathogen quantification

DNA was extracted from all swabs using PrepMan Ultra extraction reagent (Applied Biosystems), following the manufacturer’s protocols. Aliquots of DNA were diluted to 1 : 10 with UltraPure water to be used for quantitative (q)PCR assays. We performed separate qPCR assays to detect and quantify *Bd* and Rv infections against a standard curve of known pathogen concentrations, following standard methods for quantifying *Bd* zoospores [43], and Rv viral genome copies [44]. For *Bd* assays, we used serial dilutions of the Global Pandemic Lineage of *Bd* (Bd-GPL) to build a standard curve that ranged from 10^6^ zoospores at the highest concentration to 10 zoospores at the lowest. *Bd* qPCR assays were run in singlicate, and samples were designated positive if the amplification curve reached the threshold before 50 cycles. For Rv assays, we used a standard curve of frog-virus 3 (FV3), a widespread Rv lineage, that ranged from 10^7^ to 100 viral copies. Because Rv detection from swab samples can be easily confounded by environmental contamination [45], we ran Rv qPCRs in duplicate, and only considered samples positive for Rv if both replicates amplified before the forty-fifth cycle. Negative controls for all qPCR assays consisted of UltraPure water.

### Library preparation and sequencing

We sequenced bacterial communities of skin swab samples by targeting the V4 region of the bacterial 16S rRNA gene and amplifying via PCR with 515F and 806R primers [46]. The resulting amplicon products were pooled and purified using AMPure XP beads (Beckmann-Coulter) and sequenced on the Illumina Miseq platform (v2 Micro kit) at the University of Florida’s Interdisciplinary Center for Biotechnology Research (Gainesville, FL, USA), generating 2×150 paired-end reads. Raw sequence data were processed using the bioinformatics program QIIME2 [47], following the DADA2 pipeline to filter sequences for quality, remove chimeric reads, merge forward and reverse reads, and assign the final reads to amplicon sequence variants (ASVs [48]). After DADA2 processing, 2 777 550 reads remained out of a total of 2 845 465. We imported data into R with the ‘phyloseq’ package to analyse sequences [49]. In R, we reconstructed a phylogenetic tree using FastTree and assigned ASV taxonomy using the silva database classifier (version 138, accessed 21 October 2021) [50]. Any reads identified as non-bacteria, or bacteria that could not be identified at the phylum level, were excluded from analyses (a total of 205 540 reads), and the resulting ASV table was rarefied to 37 897 sequences per sample to retain all newt samples.

### Diversity metrics and statistical analyses

#### Alpha diversity

To compare the diversity of skin bacterial communities in newts, we calculated three metrics of alpha diversity for each sample: ASV richness, Shannon diversity and Faith’s phylogenetic diversity using ‘phyloseq’ in R. To determine if there were significant differences in alpha diversity metrics due to host-associated factors (mass, snout–vent length, life stage and infection type) or sampling date, we used generalized linear models (GLMs) with a Gaussian distribution, followed by ANOVA to test the significance of individual variables. Data were tested for normality using Shapiro–Wilks tests.

#### Beta diversity

To assess the beta diversity of striped newt skin bacterial communities across life stages and infection types, we calculated the Bray–Curtis dissimilarity [51] and unweighted and weighted UniFrac distances [52] using the ‘phyloseq’ package [49]. We tested for significant differences in bacterial community composition across life stages using pairwise PERMANOVAs using the ‘ecole’ package [53], then used the resulting value of the correlation coefficient (*R* ^2^) to assess how much of the community variability was explained by life stages. We ran PERMDISP models in the ‘vegan’ package [54] to verify results by testing assumptions of homogeneity of dispersion across groups. We used 999 permutations for all models, and false discovery rate (FDR)-adjusted *P*-values were generated to correct for multiple comparisons. To visualize relationships among skin bacterial communities, we generated principal coordinate analysis (PCoA) plots using the ‘phyloseq’ package.

#### Defining core bacterial communities and differential abundance analyses

It is hypothesized that hosts rely on core microbes to maintain essential functions [55], and thus identifying which life stages share members in their core skin microbiota may provide information on the function of life-stage-associated taxa. We defined the common core bacterial community for each host population (newt life stage) as the shared bacterial ASVs that were present at three different thresholds. First, we calculated all ASVs present in at least one sample within each life stage (Core-ALL). We then calculated those present in at least 75 % of samples within each life stage (Core75) and lastly those ASVs present in at least 90 % of samples within each life stage (Core90). After calculating the relative abundance of each core ASV for each population, we used the package ‘MicEco’ [56] to visualize the number and percentage of ASVs shared between any two life stages of newts via Venn diagrams. We identified bacterial families that were differentially abundant between stages using an analysis of compositions of microbiomes with bias correction (ANCOM-BC) [57] to account for variable sampling fractions (the ratio of the expected abundance of a bacterial taxon in a sample compared to the absolute abundance of that taxon in the environment) across samples. All analyses were performed in R Studio Version 4.1.1 [58].

## Results

### Sampling, pathogen qPCR and sequencing results

We sampled a total of 42 *N*. *perstriatus* individuals from two locations, during six sampling periods that spanned February 2020 to May 2021 (Table S1). Of these 42 samples, qPCR assays revealed 11 Rv-only infections, nine *Bd*-only infections, six co-infected with *Bd* and Rv, and 14 negative for both pathogens. Rv infections ranged from 117.3 to 5.3×10^8^ viral copies, while *Bd* infections ranged from 3.8 to 6.3×10^5^ zoospore copies. No infections were detected in larval stages. Metamorphosing newts exhibited higher mean *Bd* loads than other stages, while paedomorphs had far lower mean Rv loads than either adults or metamorphosing stages (Table S1). After DADA2 processing, two samples from larval newts were discarded due to low read numbers, and the remaining 40 samples were used in all downstream analyses (Table S2). Following sequence processing and assignment of reads to ASVs, we retained a total of 1 515 880 reads from striped newt samples, with a mean of 63 968 reads per sample, representing a total of 4948 unique bacterial taxa (File S1).

### Skin bacterial composition is driven by metamorphosis

**Fig. 1.**
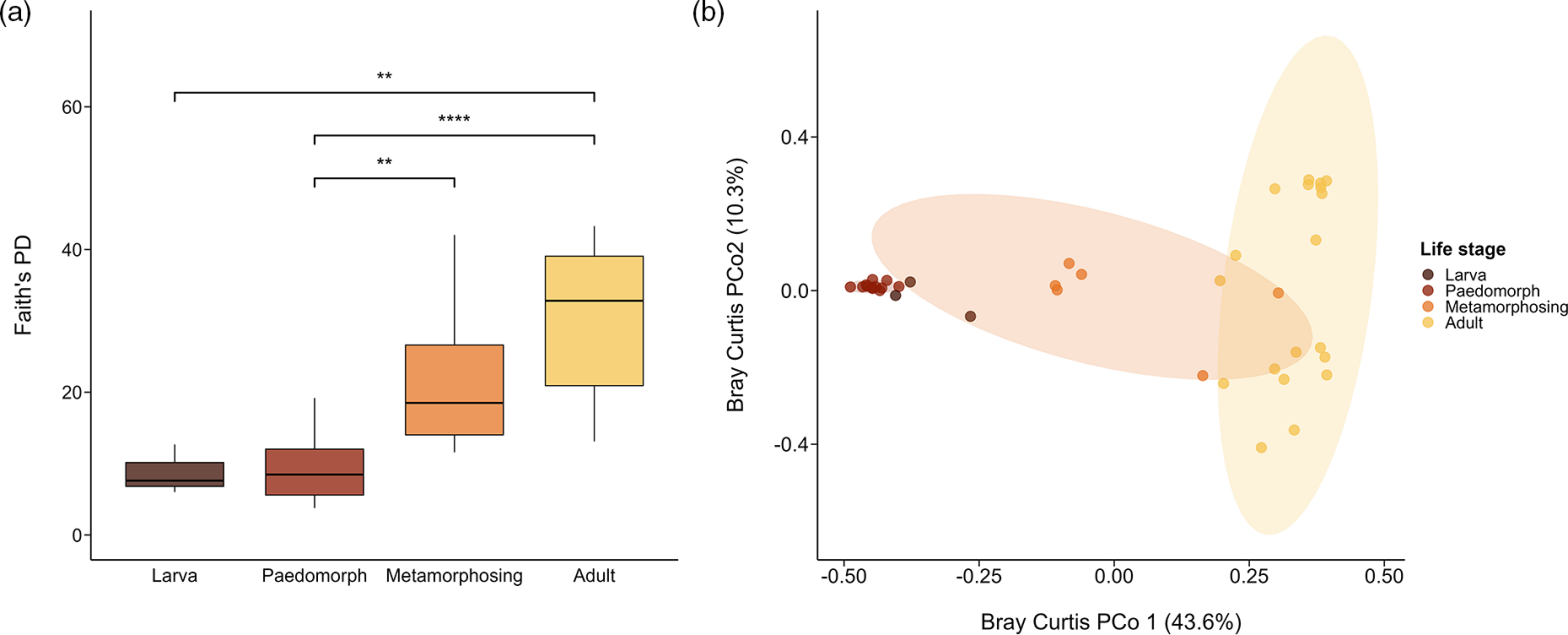
Alpha and beta metrics of bacterial diversity in *N. perstriatus* skin samples. (a) Faith’s phylogenetic diversity (PD) across life stages. Brackets indicate significant differences in alpha diversity from pairwise Wilcoxon tests (**P≤0.01, ****P≤0.005). (b) Principal coordinate analysis (PCoA) based on Bray–Curtis dissimilarity metrics across sample types. Colours indicate the life stage of each sample. Ellipses represent a 95 % confidence level for each group.

We found clear differences in alpha and beta diversity of skin bacterial communities among specific life stages as measured by ASV richness, Shannon diversity and Faith’s phylogenetic diversity (Fig. 1, Table S3). Skin bacterial communities of metamorphosing and adult newts differed from those of larvae and this was most pronounced in adults (GLM: *t*=3.93, *P*=0.00036), while there were no significant differences between larvae and paedomorphs (GLM: *t*=0.06, *P*=0.95) (Fig. 1). There was no significant effect of host body size (mass or length) or sampling date on alpha diversity (Table S3).

When considering Bray–Curtis dissimilarity, we found significant differences between all pairs of groups (PERMANOVA: adj-*P*<0.05), except between metamorphosing and larvae (PERMANOVA: adj-*P*=0.096) (Fig. 1b, Table S4). Differences were most pronounced between paedomorph and adult bacterial communities (PERMANOVA: *F*=28.27, *R* ^2^=0.49, adj-*P*=0.006) (Figs 1b and S1), but the stages differed in their within-group compositional variance (PERMDISP: *P*=0.001). PCoA plots of beta diversity metrics showed clustering of paedomorph and larval bacterial communities and little overlap with adults, while metamorphosing compositions fell between paedomorph–larva and adult clusters (Fig. S2). Unweighted UniFrac matrices showed strong significant differences between paedomorphs and adults (PERMANOVA: *R* ^2^=0.20, *F*=7.10, adj-*P*=0.006), while Weighted UniFrac matrices showed significant differences between all pairwise comparisons except between metamorphosing and larval stages (Fig. S2, Table S4). We identified significant differences in the homogeneity of dispersion between paedomorphs and adult stages for both Bray–Curtis and weighted UniFrac distances (PERMDISP: adj-*P*<0.05, Table S4).

### Bacterial diversity was not associated with pathogen infections

We did not detect any significant differences in bacterial diversity between infection groups for either alpha (GLM, ANOVA: *P*>0.05 for all models) (Table S3) or beta diversity metrics (PERMANOVA: adj-*P*>0.05 for all pairwise comparisons) (Table S5).

### Core bacteria and bacterial composition

**Fig. 2.**
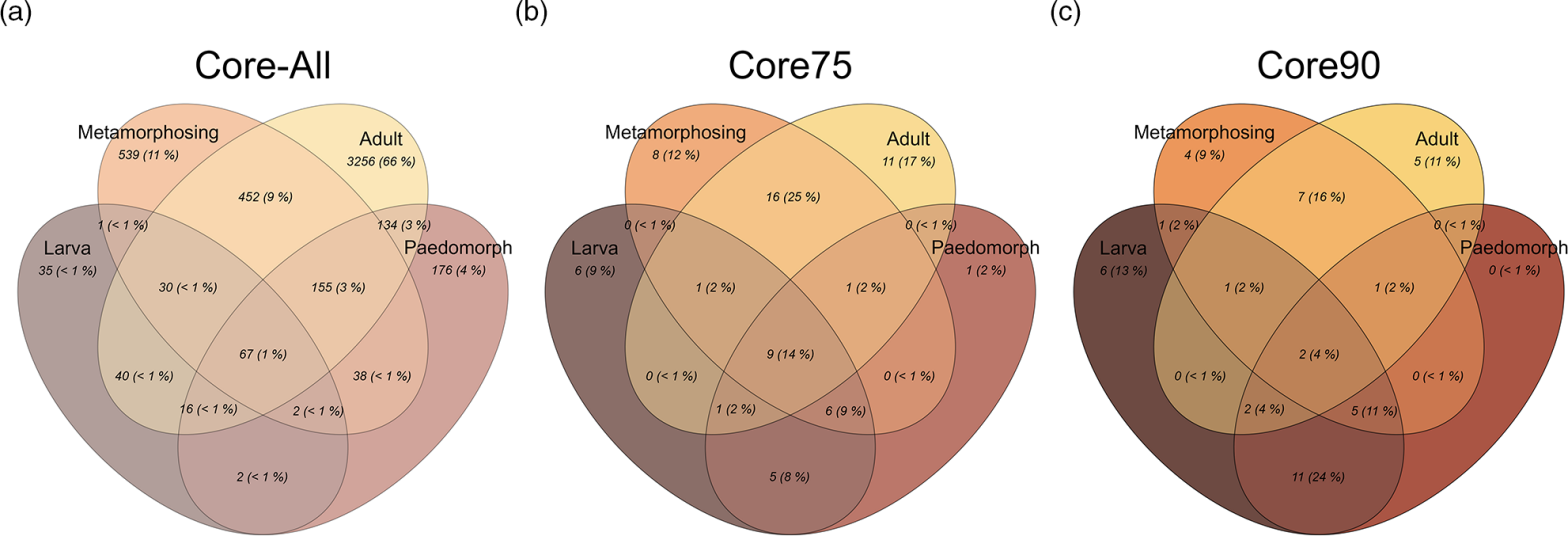
Number and proportion of shared bacterial ASVs among life stages of *N. perstriatus*. (a) All ASVs: includes all ASVs from each life stage, (b) Core75 includes ASVs present in at least 75 % of samples from each life stage, and (c) Core90 includes only those ASVs present in at least 90 % of samples from each life stage. Percentages for all three measures are calculated as the number of ASVs in each category divided by the total number of ASVs among all life stages.

In total, we detected 193 distinct ASVs in larval skin bacterial communities, 590 in paedomorphs, 1255 in metamorphosing newts and 4150 in adults (Fig. 2). Considering all individuals, 67 ASVs (1 % of the total) were shared across all stages, while this decreased to nine ASVs at the Core75 level (Fig. 2), and only two ASVs at Core90 level (Fig. 2). Adult newts had the highest number of unique ASVs overall, but they did not maintain high ASV diversity at the Core75 and Core90 levels. Metamorphosing newts exhibited the most diverse spread of Core90 ASVs, with 11/21 ASVs shared with adults, 9/21 ASVs shared with paedomorphs, 8/21 shared with larvae and 4/21 not shared with any other life stage (Fig. 2). Larva and paedomorph stages shared the highest percentage of ASVs (24 %) not shared with other stages.

To compare differences in bacteria across taxonomic levels, we compared counts at the phylum, family and genus level to determine which taxa were differentially abundant among newt life stages. In all life stages, skin bacterial communities were dominated by taxa in the phyla *Proteobacteria *, *Bacteroidota* and *Actinobacteriota* (Fig. S2). Larval, adult and metamorphosing newt bacterial communities had higher proportions of *Firmicutes* than paedomorphs, and adult and metamorphosing newts had higher proportions of *Acidobacteriota *, *Actinobacteriota* and *Verrucomicrobiota *.

**Fig. 3.**
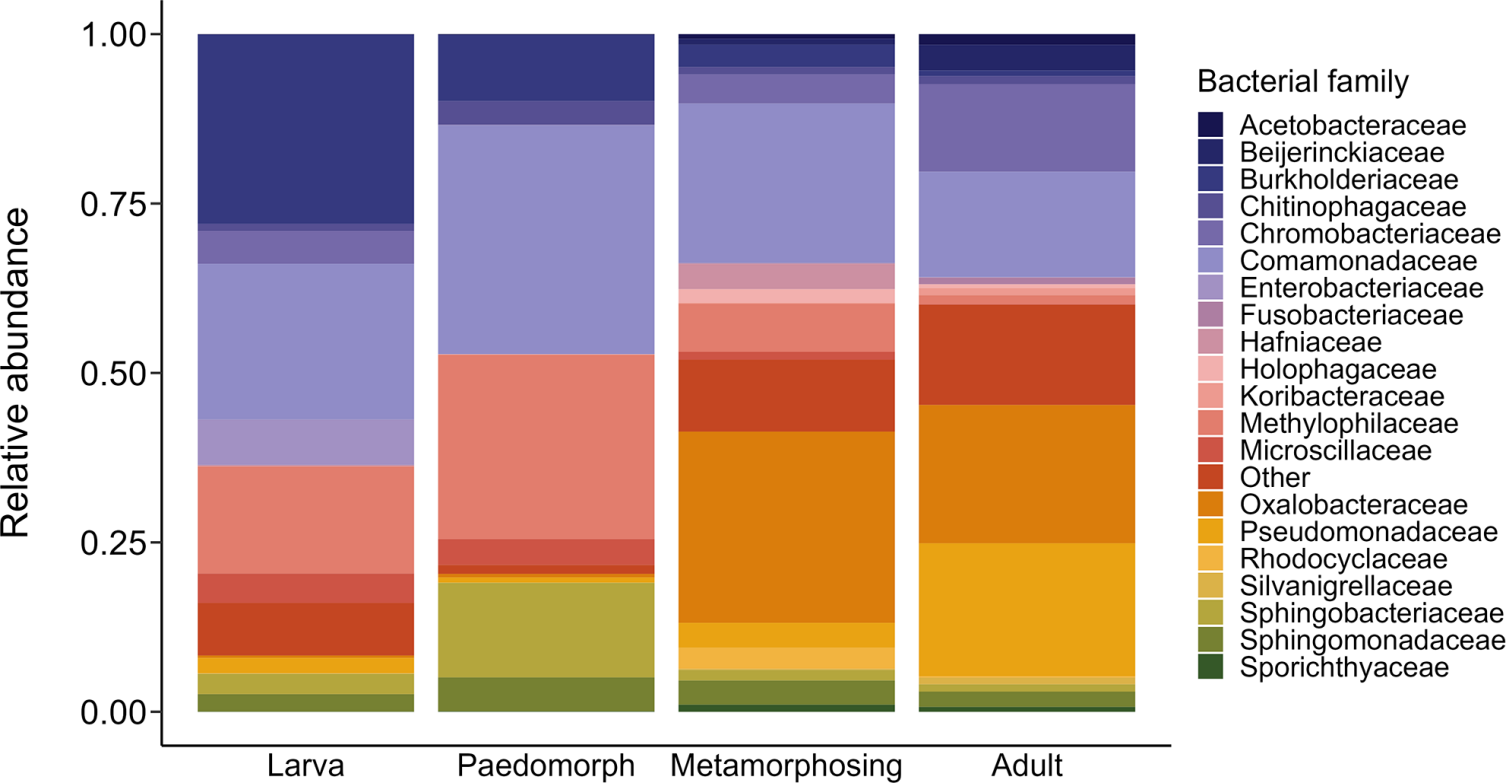
Relative abundances of bacterial families in the skin bacterial communities of *N. perstriatus*. The top 20 most abundant families are shown for each life stage, and all others are grouped and assigned to ‘Other’.

**Fig. 4.**
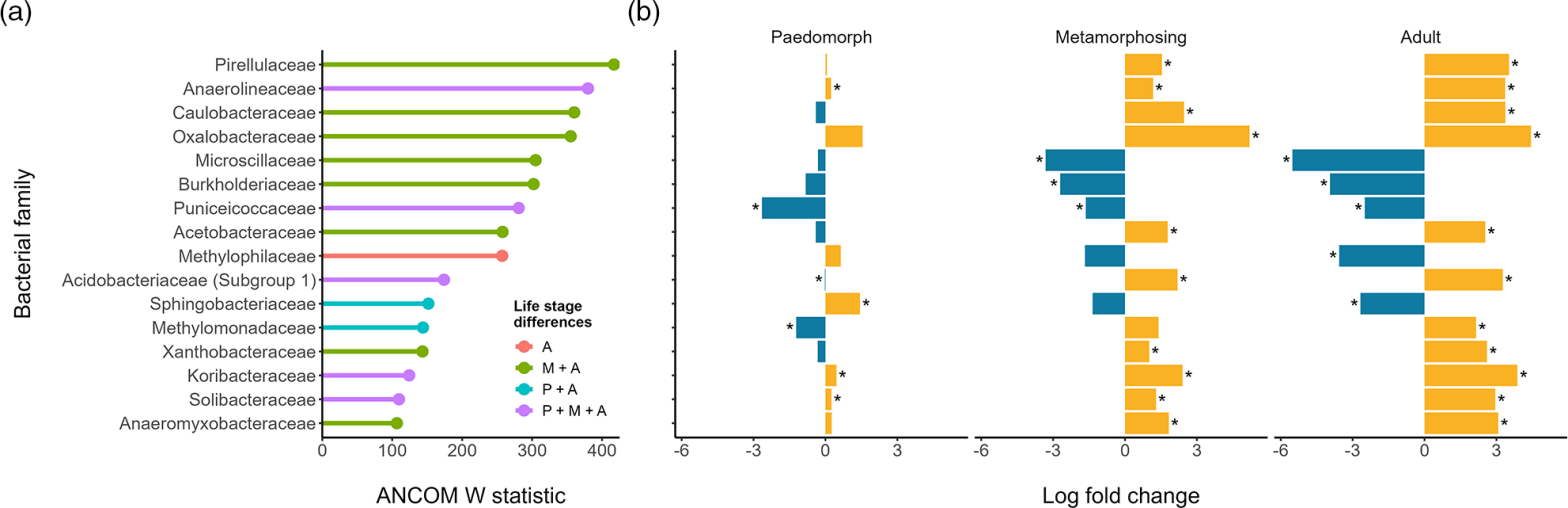
Differentially abundant bacterial families among striped newt life stages, where the ANCOM-W statistic was >100. Families are arranged in descending order by W statistic value. (a) ANCOM W statistic values for SILVA-assigned bacterial families significantly different from the larval stage. Colours indicate the life stages in which bacterial abundances differed from those of the larval stage: A = adult, P + A = paedomorph and adults, M + A = metamorphosing and adult, and P + M + A = paedomorph, metamorphosing and adult. (b) Log-fold changes in abundance of bacterial families between larvae and other life stages. Significantly different log-fold changes (adjusted-*P*≤0.05) between larvae and respective stage are indicated by an asterisk.Direction of log-fold change is colour-coded: yellow bars (right side) indicate a decrease in log fold change (depletion) of bacterial abundance, while blue bars (left side) indicate an increase in log fold change (enrichment).

At the family level, newt life stages exhibited distinct skin bacterial compositions (Fig. 3). Larvae and paedomorphs had higher proportions of *Burkholderiaceae *, *Methylophilaceae* and *Comamondaceae* (Fig. 3). Metamorphosing and adult newts had higher proportions of *Oxalobacteraceae* and *Pseudomonadaceae *. ANCOM-BC identified 135 of 257 bacterial families that were differentially abundant among life stages, but only 16 families had W-statistic values >100 (Fig. 4). Comparing the log-fold changes in abundances of these 16 families between larvae and all other stages, most were significantly different in metamorphosing (14/16) and adult (16/16) stages, with *Pirellulaceae *, *Anaerolinaeceae* and *Caulobacteraceae* having the highest W-values. Generally, most differentially abundant families were enriched (positive log-fold change) in metamorphosing and adult stages, but *Microscilliaceae*, *Burkholderiaceae* and *Puniceicoccaeceae* were significantly depleted (negative log-fold change). In contrast, the abundances of only seven bacterial families in paedomorphs differed significantly from those in larvae, with 4/16 enriched and 3/16 depleted (Fig. 4). The relative amplitude of the changes in abundances indicates that ASVs in paedomorphs were more depleted compared to metamorphosing and adult stages (Fig. 4).

## Discussion

Here we investigated skin bacterial communities across life stages of the striped newt, an amphibian with a complex life cycle that involves variable ontogenetic strategies [59]. This species exhibits facultatively paedomorphic development routes in which paedomorphic individuals undergo metamorphosis while also reproducing, resulting in spatiotemporal overlap of pre-, mid- and post-metamorphic stages [42]. Because of this ontogenetic overlap and gradual metamorphosis, we were able to compare not only differences among newt life stages but also the shift in bacterial composition during metamorphosis. Furthermore, by comparing the skin bacterial communities of different life stages occupying the same aquatic habitat we were able to infer host-associated influences on skin microbiome composition through ontogeny without the confounding effects of habitat differences. Overall, we found indirect support that environment and host development can drive the structure of the amphibian microbiome through time. We discuss the potential mechanisms for these changes and highlight the importance of our assessment as a way to disentangle microbial dynamics in declining species.

### Bacterial communities shift with ontogeny

Many studies have shown that the host-associated microbiota of amphibians shifts across ontogeny [11, 14, 35, 60]. Most of these studies focus on anuran species that transition from aquatic larvae to terrestrial post-metamorphic stages [35], making it difficult to tease apart the effects of host development and environmental influence on skin microbial assembly. In some species where life stages spatiotemporally overlap in aquatic habitats, different life stages harbour distinct skin bacterial communities, indicating that microbial assembly is host-linked and driven by ontogeny [16, 61].

Our examination of the skin bacterial diversity in coexisting aquatic *N. perstriatus* life stages shows that bacterial communities shift during ontogeny, and that richness increased during and after metamorphosis (Fig. 1). Substantial immunological and structural changes occur in the skin during metamorphosis [17, 18], and thus developmental shifts in immunity or skin structure may be the mechanisms that drive the assembly of skin bacterial communities. We found that larval newts had the lowest abundances of skin microbes and lower diversity across all metrics (Fig. 1), which may indicate the importance of neutral processes, such as ecological drift and dispersal limitation, in driving early microbial assembly [62]. Conversely, low diversity may indicate strong host selection, though typically microbial diversity decreases with ontogeny as the role of host selection in filtering the microbial community increases [63]. Paedomorphs had the second lowest diversity and bacterial communities were not significantly different from larvae for most diversity metrics, though both were distinct from metamorphosing and adult newts. Though our sample size of larvae was small, they did not differ significantly in any diversity metric from paedomorphs. This suggests that a similar mechanism may drive the microbial assembly of both stages and recruit largely the same taxa.

We found higher bacterial diversity in metamorphosing and adult *N. perstriatus* compared to larval and paedomorphic stages, as the gradual restructuring of skin [24] and immune function [17] during metamorphosis probably selects for different associated microbial taxa [13]. Since we were able to compare the skin bacterial communities of newts during metamorphosis, we found that metamorphosing newts had transitional bacterial communities consisting of the dominant taxa of paedomorphic and post-metamorphic adults, and metrics of bacterial diversity and abundance that fell somewhere between pre- and post-metamorphic stages (Figs 1 and 2). This indicates that the shift in skin bacterial communities between pre- and post-metamorphic stages coincides with changes in skin structure and immune function during metamorphosis. However, our sample sizes of larvae and metamorphosing newts were smaller than both adults and paedomorphs, so we probably observed a much more limited range of bacterial compositions present within these stages.

Consistent with other studies, post-metamorphic adult newts had the highest bacterial diversity [61, 64, 65] and the most distinct abundances of bacterial families but retained very few unique ASVs at the common Core90 level. Adult *N. perstriatus* are primarily terrestrial and make seasonal migrations to breeding ponds, while all pre-metamorphic and metamorphosing stages are strictly aquatic; therefore, the increased diversity and compositional variability in adult skin bacteria could be from terrestrial or soil-associated taxa that are present in low abundances. It is likely that migrations between aquatic and terrestrial habitats incur predictable shifts in the composition of the skin microbial community, as seasonality has been shown to drive microbial composition in other species [16, 36]. However, in *N. perstriatus* these stages all inhabit the same aquatic environment during the same times yet possess distinct bacterial communities, suggesting that host selection is a primary driver of bacterial assembly for this species.

### Pathogen infection not associated with shifts in bacterial composition

We did not find support for pathogen infection influencing the composition of the striped newt skin microbiome possibly because our sample sizes were small and uneven among life stages and between pathogens. Microbial interactions, including pathogen invasion, are known to alter community assembly though facilitative, competitive and mutualistic relationships that can recruit, inhibit or exclude certain taxa [66, 67]. Previous works have shown that *Bd* [38, 68, 69] and Rv [70] infections can disrupt skin microbial composition, but the level of microbial disturbance is dependent on infection load [68]. Since we examined only infection status, as *Bd* and Rv loads in our samples ranged over several orders of magnitude across different life stages, any effects of increasing infection load on bacterial diversity were probably masked by other factors, such as metamorphosis. With larger sample sizes of life stage and infection groups, we could compare relationships between different pathogen load and bacterial composition and examine if these relationships are constant through ontogeny.

*N. perstriatus* newts are susceptible to both *Bd* and Rv but mortalities have only been attributed to Rv in experimental and natural conditions [41, 71]. Periods of microbial restructuring, such as those that occur during metamorphosis, may accompany or exacerbate lapses in host immunological function [13]. We found higher *Bd* loads in metamorphosing newts, and prior documented mortalities were observed primarily in this stage; thus, we hypothesize that metamorphosis-induced shifts of the skin microbiomes may impair microbially dependent host functions, leaving metamorphosing stages more susceptible to pathogen infection. Alternatively, the depleted bacterial communities of pre-metamorphic newts may indicate a lack of skin microbial defences.

While we had too few co-infections to meaningfully assess impacts on skin bacterial diversity, the global overlap of *Bd* and Rv in amphibians heightens the need to understand how ontogenetic shifts of host-associated microbial communities might interact with multiple invasive pathogens to influence emerging diseases. Susceptibility to infection varies among amphibian life stages [37, 72], and has been linked to shifts in the microbiome [36, 70]. However, most studies of pathogen–microbiome associations through development focus on a single focal pathogen, and future work should incorporate the effects of multi-pathogen infections on the microbiome.

### Management implications for *N. perstriatus*

Severe population decline throughout the range of *N. perstriatus* has prompted a captive breeding programme to restore newts to areas where they were previously abundant [40]. Despite the release of thousands of newts over a 10-year period, recruitment remains low and populations have not recovered [73]. Currently, captively reared *N. perstriatus* are mainly released at the paedomorph-to-metamorphosing transition, as this stage takes only a year to develop and is sexually mature. However, captive newts are not head-started or acclimated in semi-natural conditions before release, which has been shown to buffer the impacts of novel stressors and increase survivorship of released individuals across a variety of taxa [74–76]. Our findings show that metamorphosing stages exhibit transitional microbiomes during a time of physiological stress, which may indicate a lapse in host functions. Additionally, many studies show that captively reared amphibians exhibit depleted microbiomes compared to wild counterparts [4, 77, 78], potentially impairing biological functions essential for host health [79–81]. Releases of captive-bred newts at the metamorphosing stages may not be the most optimal strategy if these stages are experiencing physiological stress while also exhibiting depleted, and possibly functionally impaired, microbiomes.

We recommend conservation managers consider soft-release strategies for the striped newt, such as natural mesocosm rearing, to both acclimate newts to environmental stressors and ameliorate the microbiome before release to prepare animals for the stressful transition from captive to natural conditions. Adaptive management strategies for ‘rewilding’ the microbiome via soft-releases have been shown to increase body condition and antifungal properties for captively reared amphibians [81, 82], and should be considered for *N. perstriatus* conservation planning. Although we found no correlation between newt infection status and skin bacterial diversity, *Bd* and Rv are enzootic in these environments and present a constant threat to captive release success. We also recommend the implementation of varied life stages for release, specifically of post-metamorphic adults, which typically have more robust immune function than other stages [17]. We acknowledge the limited space and funding of many captive breeding facilities [83] and the challenges this presents to maintaining amphibians for longer periods of time and through changes in habitat requirements. However, given the lack of establishment of released striped newts, a shift in rearing or release strategies may be necessary to ensure successful repatriation.

## Conclusions

Overall, we have demonstrated that developmental shifts are important factors influencing skin bacterial communities in paedomorphic salamanders. Despite small sample sizes for some stages, differences in bacterial diversity were greatest during and after metamorphosis, indicating that the restructuring of bacterial communities and host physiology during metamorphosis is synchronous. Because the different developmental stages overlap spatiotemporally, the shift in bacterial composition from aquatic to post-metamorphic-associated taxa begins before hosts leave aquatic environments, indicating that host selection probably plays a major role in the microbial assembly. The evolution of paedomorphosis in salamanders may provide a prolonged period for microbial structuring during the post-larval period compared to typical larva-to-metamorph strategies, potentially providing a more stabilized community before the onset of metamorphosis. Specifically, the continued period of microbial structuring may insulate paedomorphs from functional disruption during these periods compared to larvae. The timing of metamorphosis in amphibians can be triggered by environmental or density-dependent stressors [21, 84, 85] that might disrupt microbiomes until reaching new alternative stable states [86]. Future research should further quantify the temporal dynamics of microbial communities in the skin and environment to examine how periods of microbial restructuring impact immune function and microbial colonization, especially of pathogens, during different developmental strategies.

## Supplementary Data

Supplementary material 1

## References

[1] Hooper LV, Macpherson AJ (2010). Immune adaptations that maintain homeostasis with the intestinal microbiota. Nat Rev Immunol.

[2] Kueneman JG, Woodhams DC, Van Treuren W, Archer HM, Knight R (2016). Inhibitory bacteria reduce fungi on early life stages of endangered Colorado boreal toads (*Anaxyrus boreas*). ISME J.

[3] Hill AJ, Leys JE, Bryan D, Erdman FM, Malone KS (2018). Common cutaneous bacteria isolated from snakes inhibit growth of *Ophidiomyces ophiodiicola*. EcoHealth.

[4] Ross AA, Rodrigues Hoffmann A, Neufeld JD (2019). The skin microbiome of vertebrates. Microbiome.

[5] Nava-González B, Suazo-Ortuño I, López PB, Maldonado-López Y, Lopez-Toledo L (2021). Inhibition of *Batrachochytrium dendrobatidis* infection by skin bacterial communities in wild amphibian populations. Microb Ecol.

[6] Lauer A, Simon MA, Banning JL, André E, Duncan K (2007). Common cutaneous bacteria from the eastern red-backed salamander can inhibit pathogenic fungi. Copeia.

[7] Flechas SV, Sarmiento C, Cárdenas ME, Medina EM, Restrepo S (2012). Surviving chytridiomycosis: differential anti-*Batrachochytrium dendrobatidis* activity in bacterial isolates from three lowland species of *Atelopus*. PLoS One.

[8] Colombo BM, Scalvenzi T, Benlamara S, Pollet N (2015). Microbiota and mucosal immunity in amphibians. Front Immunol.

[9] Becker MH, Walke JB, Cikanek S, Savage AE, Mattheus N (2015). Composition of symbiotic bacteria predicts survival in Panamanian golden frogs infected with a lethal fungus. Proc Biol Sci.

[10] Douglas AJ, Hug LA, Katzenback BA (2021). Composition of the North American wood frog (*Rana sylvatica*) bacterial skin microbiome and seasonal variation in community structure. Microb Ecol.

[11] Griffiths SM, Harrison XA, Weldon C, Wood MD, Pretorius A (2018). Genetic variability and ontogeny predict microbiome structure in a disease-challenged montane amphibian. ISME J.

[12] Kohl KD, Cary TL, Karasov WH, Dearing MD (2013). Restructuring of the amphibian gut microbiota through metamorphosis. Environ Microbiol Rep.

[13] Bataille A, Lee-Cruz L, Tripathi B, Waldman B (2018). Skin bacterial community reorganization following metamorphosis of the fire-bellied toad (*Bombina orientalis*). Microb Ecol.

[14] Yang H, Liu R, Meng J, Wang H (2020). Changes in intestinal microbial community of *Rana chensinensis* tadpoles during metamorphosis. Aquaculture.

[15] Fontaine SS, Mineo PM, Kohl KD (2021). Changes in the gut microbial community of the eastern newt (*Notophthalmus viridescens*) across its three distinct life stages. FEMS Microbiol Ecol.

[16] Martínez-Ugalde E, Ávila-Akerberg V, González Martínez TM, Vázquez Trejo M, Zavala Hernández D (2022). The skin microbiota of the axolotl *Ambystoma altamirani* is highly influenced by metamorphosis and seasonality but not by pathogen infection. Anim Microbiome.

[17] Rollins-Smith LA (1998). Metamorphosis and the amphibian immune system. Immunol Rev.

[18] Brown DD, Cai L (2007). Amphibian metamorphosis. Dev Biol.

[19] Hernández-Gómez O, Hua J (2023). From the organismal to biosphere levels: environmental impacts on the amphibian microbiota. FEMS Microbiol Rev.

[20] Duellman WE, Trueb L (1994). Biology of Amphibians.

[21] Whiteman HH (1994). Evolution of facultative paedomorphosis in salamanders. Q Rev Biol.

[22] Wiens JJ, Bonett RM, Chippindale PT, Anderson F (Andy) (2005). Ontogeny discombobulates phylogeny: paedomorphosis and higher-level salamander relationships. Syst Biol.

[23] Fox H (1985). Changes in amphibian skin during larval development and metamorphosis. Metamorphosis.

[24] Ohmura H, Wakahara M (1998). Transformation of skin from larval to adult types in normally metamorphosing and metamorphosis-arrested salamander, *Hynobius retardatus*. Differentiation.

[25] Johnson CK, Voss SR, Shi Y-B (2013). Current Topics in Developmental Biology.

[26] Haislip NA, Gray MJ, Hoverman JT, Miller DL (2011). Development and disease: how susceptibility to an emerging pathogen changes through anuran development. PLoS One.

[27] Green DE, Converse KA, Schrader AK (2002). Epizootiology of sixty-four amphibian morbidity and mortality events in the USA, 1996-2001. Ann N Y Acad Sci.

[28] Johnson PTJ, Kellermanns E, Bowerman J (2011). Critical windows of disease risk: amphibian pathology driven by developmental changes in host resistance and tolerance. Funct Ecol.

[29] Savage AE, Sredl MJ, Zamudio KR (2011). Disease dynamics vary spatially and temporally in a North American amphibian. Biol Conserv.

[30] Longo AV, Ossiboff RJ, Zamudio KR, Burrowes PA (2013). Lability in host defenses: terrestrial frogs die from chytridiomycosis under enzootic conditions. J Wildl Dis.

[31] Brunner JL, Storfer A, Gray MJ, Hoverman JT (2015). Ranaviruses.

[32] Olori JC, Netzband R, McKean N, Lowery J, Parsons K (2018). Multi-year dynamics of ranavirus, chytridiomycosis, and co-infections in a temperate host assemblage of amphibians. Dis Aquat Org.

[33] Hall EM, Goldberg CS, Brunner JL, Crespi EJ (2018). Seasonal dynamics and potential drivers of ranavirus epidemics in wood frog populations. Oecologia.

[34] Thumsová B, Price SJ, González-Cascón V, Vörös J, Martínez-Silvestre A (2022). Climate warming triggers the emergence of native viruses in Iberian amphibians. iScience.

[35] Kueneman JG, Parfrey LW, Woodhams DC, Archer HM, Knight R (2014). The amphibian skin-associated microbiome across species, space and life history stages. Mol Ecol.

[36] Longo AV, Savage AE, Hewson I, Zamudio KR (2015). Seasonal and ontogenetic variation of skin microbial communities and relationships to natural disease dynamics in declining amphibians. R Soc Open Sci.

[37] Langhammer PF, Burrowes PA, Lips KR, Bryant AB, Collins JP (2014). Susceptibility to the amphibian chytrid fungus varies with ontogeny in the direct-developing frog, *Eleutherodactylus coqui*. J Wildl Dis.

[38] Longo AV, Zamudio KR (2017). Environmental fluctuations and host skin bacteria shift survival advantage between frogs and their fungal pathogen. ISME J.

[39] Campbell LJ, Pawlik AH, Harrison XA, Lesbarrères D (2020). Amphibian ranaviruses in Europe: important directions for future research. FACETS.

[40] Farmer AL, Enge KM, Jensen JB, Stevenson DJ, Smith LL (2017). A range-wide assessment of the status and distribution of the striped newt (*Notophthalmus perstriatus*). Herpetol Conserv Biol.

[41] Hartmann AM, Maddox ML, Ossiboff RJ, Longo AV (2022). Sustained ranavirus outbreak causes mass mortality and morbidity of imperiled amphibians in Florida. Ecohealth.

[42] Johnson SA (2002). Life history of the striped newt at a north-central Florida breeding pond. Southeastern Naturalist.

[43] Boyle DG, Boyle DB, Olsen V, Morgan JAT, Hyatt AD (2004). Rapid quantitative detection of chytridiomycosis (*Batrachochytrium dendrobatidis*) in amphibian samples using real-time Taqman PCR assay. Dis Aquat Organ.

[44] Allender MC, Bunick D, Mitchell MA (2013). Development and validation of TaqMan quantitative PCR for detection of frog virus 3-like virus in eastern box turtles (*Terrapene carolina carolina*). J Virol Methods.

[45] Miller DL, Pessier AP, Hick P, Whittington RJ, Gray MJ, Chinchar VG (2015). Ranaviruses.

[46] Caporaso JG, Lauber CL, Walters WA, Berg-Lyons D, Lozupone CA (2011). Global patterns of 16S rRNA diversity at a depth of millions of sequences per sample. Proc Natl Acad Sci U S A.

[47] Bolyen E, Rideout JR, Dillon MR, Bokulich NA, Abnet CC (2019). Reproducible, interactive, scalable and extensible microbiome data science using QIIME 2. Nat Biotechnol.

[48] Callahan BJ, McMurdie PJ, Rosen MJ, Han AW, Johnson AJA (2016). DADA2: High-resolution sample inference from Illumina amplicon data. Nat Methods.

[49] McMurdie PJ, Holmes S (2013). phyloseq: an R package for reproducible interactive analysis and graphics of microbiome census data. PLoS One.

[50] Quast C, Pruesse E, Yilmaz P, Gerken J, Schweer T (2013). The SILVA ribosomal RNA gene database project: improved data processing and web-based tools. Nucleic Acids Res.

[51] Bray JR, Curtis JT (1957). An ordination of the upland forest communities of Southern Wisconsin. Ecol Monog.

[52] Lozupone C, Lladser ME, Knights D, Stombaugh J, Knight R (2011). UniFrac: an effective distance metric for microbial community comparison. ISME J.

[53] Smith R (2021). ecole: ecole: School of Ecology Package. https://github.com/phytomosaic/ecole.

[54] Oksanen J, Blanchet FG, Kindt R, Legendre P, Minchin PR (2013). Package ‘Vegan'. Community Ecology Package, Version.

[55] Neu AT, Allen EE, Roy K (2021). Defining and quantifying the core microbiome: challenges and prospects. Proc Natl Acad Sci U S A.

[56] Russel J (2022). MicEco: various functions for microbial community data.

[57] Lin H, Peddada SD (2020). Analysis of compositions of microbiomes with bias correction. Nat Commun.

[58] R Core Team (2021). R: A Language and Environment for Statistical Computing. Vienna, Austria: R Foundation for Statistical Computing. https://www.R-project.org/.

[59] Dodd CK (1993). Cost of living in an unpredictable environment: the ecology of striped newts *Notophthalmus perstriatus* during a prolonged drought. Copeia.

[60] Albecker MA, Belden LK, McCoy MW (2019). Comparative analysis of anuran amphibian skin microbiomes across Inland and Coastal Wetlands. Microb Ecol.

[61] Michaels CJ (2022). Effects of aquatic and terrestrial habitats on the skin microbiome and growth rate of juvenile alpine newts *Ichthyosaura alpestris*. Herpetol J.

[62] Keady MM, Jimenez RR, Bragg M, Wagner JCP, Bornbusch SL (2023). Ecoevolutionary processes structure milk microbiomes across the mammalian tree of life. Proc Natl Acad Sci U S A.

[63] Heys C, Cheaib B, Busetti A, Kazlauskaite R, Maier L (2020). Neutral processes dominate microbial community assembly in Atlantic Salmon, *Salmo salar*. Appl Environ Microbiol.

[64] Sabino-Pinto J, Galán P, Rodríguez S, Bletz MC, Bhuju S (2017). Temporal changes in cutaneous bacterial communities of terrestrial- and aquatic-phase newts (Amphibia). Environ Microbiol.

[65] Wuerthner VP, Hua J, Hernández-Gómez O (2022). Life stage and proximity to roads shape the skin microbiota of eastern newts (*Notophthalmus viridescens*). Environ Microbiol.

[66] Voyles J, Rosenblum EB, Berger L (2011). Interactions between *Batrachochytrium dendrobatidis* and its amphibian hosts: a review of pathogenesis and immunity. Microbes Infect.

[67] Debray R, Herbert RA, Jaffe AL, Crits-Christoph A, Power ME (2022). Priority effects in microbiome assembly. Nat Rev Microbiol.

[68] Jani AJ, Briggs CJ (2014). The pathogen *Batrachochytrium dendrobatidis* disturbs the frog skin microbiome during a natural epidemic and experimental infection. Proc Natl Acad Sci U S A.

[69] Walke JB, Becker MH, Loftus SC, House LL, Teotonio TL (2015). Community structure and function of amphibian skin microbes: an experiment with bullfrogs exposed to a Chytrid fungus. PLoS One.

[70] Harrison XA, Price SJ, Hopkins K, Leung WTM, Sergeant C (2019). Diversity-stability dynamics of the amphibian skin microbiome and susceptibility to a lethal viral pathogen. Front Microbiol.

[71] Means RC, Means RPM, Beshel M, Mendyk R, Hill P (2016). A conservation strategy for the imperiled Western striped newt in the Apalachicola National forest. FL.

[72] Hoverman JT, Gray MJ, Haislip NA, Miller DL (2011). Phylogeny, life history, and ecology contribute to differences in amphibian susceptibility to ranaviruses. Ecohealth.

[73] Means RC, Means RPM, Beshel M, Mendyk R, Hill P (2017). A conservation strategy for the imperiled Western striped newt in the Apalachicola National Forest. FL.

[74] Kenison EK, Williams RN (2018). Training for translocation: predator conditioning induces behavioral plasticity and physiological changes in captive Eastern Hellbenders (*Cryptobranchus alleganiensis alleganiensis*) (Cryptobranchidae, Amphibia). Diversity.

[75] Teixeira CP, de Azevedo CS, Mendl M, Cipreste CF, Young RJ (2007). Revisiting translocation and reintroduction programmes: the importance of considering stress. Animal Behaviour.

[76] Dickens MJ, Delehanty DJ, Michael Romero L (2010). Stress: an inevitable component of animal translocation. Biol Conserv.

[77] Becker MH, Richards-Zawacki CL, Gratwicke B, Belden LK (2014). The effect of captivity on the cutaneous bacterial community of the critically endangered Panamanian golden frog (Atelopus zeteki). Biol Conserv.

[78] Bataille A, Lee-Cruz L, Tripathi B, Kim H, Waldman B (2016). Microbiome variation across amphibian skin regions: implications for Chytridiomycosis mitigation efforts. Microb Ecol.

[79] Sommer F, Bäckhed F (2013). The gut microbiota--masters of host development and physiology. Nat Rev Microbiol.

[80] Dallas JW, Warne RW (2023). Captivity and animal microbiomes: potential roles of microbiota for influencing animal conservation. Microb Ecol.

[81] Kueneman JG, Bletz MC, Becker M, Gratwicke B, Garcés OA (2022). Effects of captivity and rewilding on amphibian skin microbiomes. Biol Conserv.

[82] Estrada A, Medina D, Gratwicke B, Ibáñez R, Belden LK (2022). Body condition, skin bacterial communities and disease status: insights from the first release trial of the limosa harlequin frog, Atelopus limosus. Proc Biol Sci.

[83] Della Togna G, Howell LG, Clulow J, Langhorne CJ, Marcec-Greaves R (2020). Evaluating amphibian biobanking and reproduction for captive breeding programs according to the amphibian conservation action Plan objectives. Theriogenology.

[84] Semlitsch RD, Wilbur HM (1988). Effects of pond drying time on metamorphosis and survival in the Salamander *Ambystoma talpoideum*. Copeia.

[85] Relyea RA (2007). Getting out alive: how predators affect the decision to metamorphose. Oecologia.

[86] Relman DA (2012). The human microbiome: ecosystem resilience and health. Nutr Rev.

