# Supplementary material for: Ontogeny drives shifts in skin bacterial communities in facultatively paedomorphic salamanders
Source: Microbiology (Reading). 2023 Oct 10;169(10):001399. doi: 10.1099/mic.0.001399 (PMC10634365; doi:10.1099/mic.0.001399)
Supplement: Supplementary material 1 [file mic-169-1399-s001.pdf]

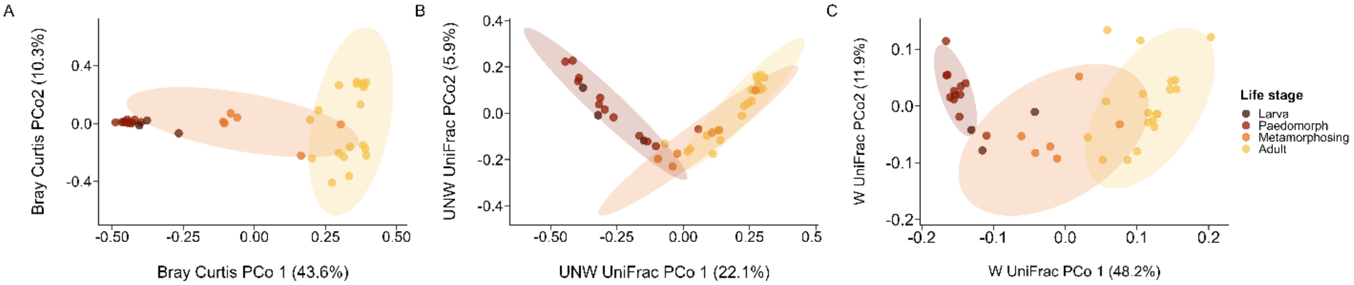

Supplemental Figure 1. Beta diversity metrics of skin bacterial communities across life stages and infection types in *N. perstriatus*. Shapes represent different infection types, while life stages are coded by color. The ellipses are drawn and shaded by life stage, except for larva where points were too few.

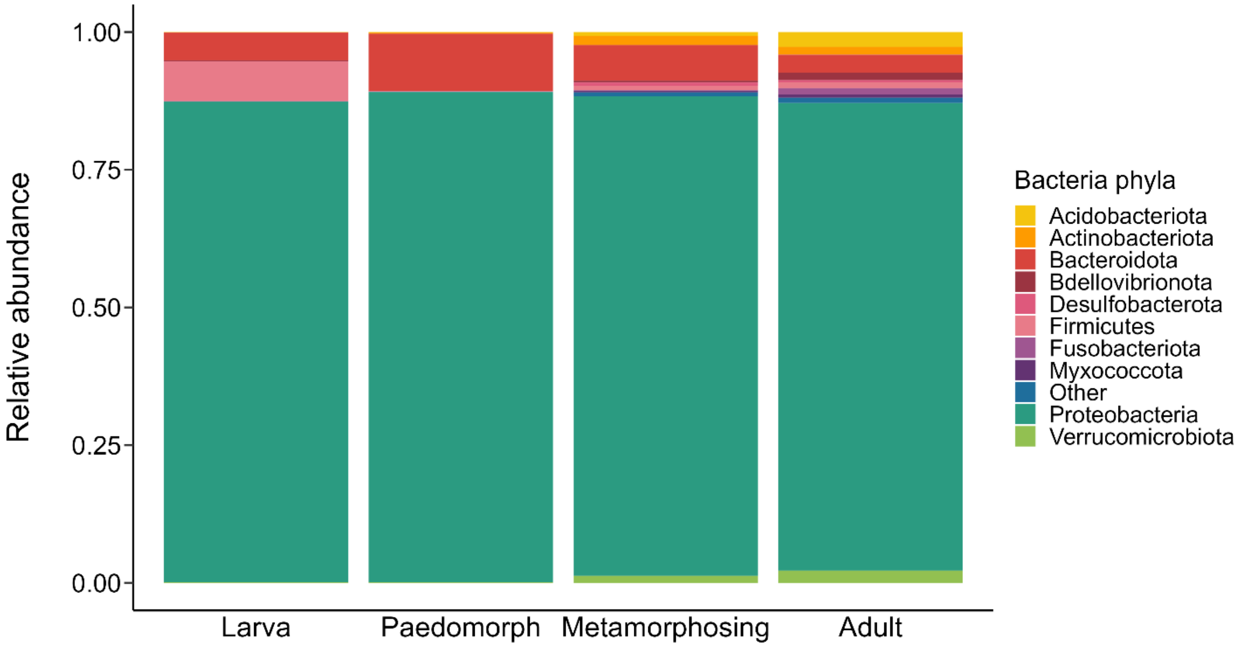

Supplemental Figure 2. Relative abundances of bacterial phyla in the skin of *N. perstriatus* among life stages. The top 10 most abundant phyla for each life stage are shown, while the rest are assigned as "Other".

Supplementary Table 1. Summary table of all samples used in analyses. Sample ID represents an individual newt sample with a respective SRA Biosample numbers and unique Barcode sequence. Survey is the month and year newt samples were collected. Pond indicates which of the two wetlands the newts were sampled from. Mass is the weight of each animal in grams, and SVL is snout-vent-length of each individual.

Sample ID	Biosample No.	Barcode sequence	Survey	Pond	Mass	SVL
SN187	1_SN187_515rcbc00	AGCCTTCGTCGC	February 2020	1	2.65	3.73
SN192	2_SN192_515rcbc12	CGTATAAATGCG	February 2020	1	2.75	3.81
SN193	3_SN193_515rcbc24	TGACTAATGGCC	February 2020	1	2.25	3.62
SN198	4_SN198_515rcbc36	GTGGAGTCTCAT	February 2020	1	1.55	3.2
SN210	5_SN210_515rcbc48	TGATGTGCTAAG	February 2020	1	1.2	3.03
SN211	6_SN211_515rcbc60	TGTGCACGCCAT	February 2020	1	1.7	3.5
SN212	7_SN212_515rcbc72	GGTGAGCAAGCA	February 2020	1	2.2	3.83
SN213	8_SN213_515rcbc84	CTATGTATTAGT	February 2020	1	2	3.58
SN214	9_SN214_515rcbc01	TCCATACCGGAA	February 2020	1	2.55	3.8
SN215	10_SN215_515rcbc13	ATGCTGCAACAC	February 2020	1	2.05	3.7
SN363	11_SN363_515rcbc25	CGGGACACCCGA	March 2020	1	2.2	3.39
SN364	12_SN364_515rcbc37	ACCTTACACCTT	March 2020	1	1.65	3.49
SN365	13_SN365_515rcbc49	GTAGTAGACCAT	March 2020	1	2.55	3.78
SN369	14_SN369_515rcbc61	CCGGACAAGAAG	March 2020	1	2	3.54
SN895	15_SN895_515rcbc73	TAAATATACCCT	February 2021	1	1.35	2.92
SN896	16_SN896_515rcbc85	ACTCCCGTGTGA	February 2021	1	1.3	2.88
SN897	17_SN897_515rcbc02	AGCCCTGCTACA	February 2021	1	2.7	3.85
SN898	18_SN898_515rcbc14	ACTCGCTCGCTG	February 2021	1	1.25	2.82
SN899	19_SN899_515rcbc26	CTGTCTATACTA	February 2021	1	1.4	3
SN903	20_SN903_515rcbc38	TAATCTCGCCGG	February 2021	1	1.35	2.81
SN907	21_SN907_515rcbc50	AGTAAAGATCGT	February 2021	1	1.4	3.19
SN908	22_SN908_515rcbc62	TTGCTGGACGCT	February 2021	1	1.45	3.14
SN911	23_SN911_515rcbc74	TTGCGGACCCTA	February 2021	1	1.65	3.22
SN942	24_SN942_515rcbc86	CGGTATAGCAAT	March 2021	2	2.15	3.68
SN943	25_SN943_515rcbc03	CCTAACGGTCCA	March 2021	2	1.75	3.31
SN944	26_SN944_515rcbc15	TTCCTTAGTAGT	March 2021	2	1.5	2.96
SN957	27_SN957_515rcbc27	TATGCCAGAGAT	March 2021	2	1.7	3.27
SN988	28_SN988_515rcbc39	ATCTAGTGGCAA	March 2021	1	1.6	3.44
SN989	29_SN989_515rcbc51	CTCGCCCTCGCC	March 2021	1	2.1	3.52
SN990	30_SN990_515rcbc63	TACTAACGCGGT	March 2021	1	1.6	3.13
SN992	31_SN992_515rcbc75	GTCGTCCAAATG	March 2021	1	1.45	2.77
SN993	32_SN993_515rcbc87	GACTCTGCTCAG	March 2021	1	1.5	3.34
SN994	33_SN994_515rcbc04	CGCGCCTTAAAC	March 2021	1	1.5	3.42
SN995	34_SN995_515rcbc16	CGTCCGTATGAA	March 2021	1	1.5	2.96
SN1014	35_SN1014_515rcbc28	CGTTTGGAATGA	April 2021	2	1.45	2.99
SN1015	36_SN1015_515rcbc40	ACGCTTAACGAC	April 2021	2	1.2	3
SN1052	38_SN1052_515rcbc64	GCGATCACACCT	April 2021	2	1.65	3.17
SN1060	39_SN1060_515rcbc76	TGCACAGTCGCT	May 2021	1	1	2.2
SN1061	40_SN1061_515rcbc88	GTCATGCTCCAG	May 2021	1	1.2	2.4
SN1062	41_SN1062_515rcbc05	TATGGTACCCAG	May 2021	1	1.2	2.4
SN1066	42_SN1066_515rcbc17	ACGTGAGGAACG	May 2021	1	0.9	2.1

Supplementary Table 2 Description of samples used in diversity analyses. Numbers in bold indicate the total number of samples representing each life stage (column) or infection type (row). Mean infection loads and ± standard errors are estimated pathogen copies from qPCR assays for each infected life stage group and includes single and coinfected samples.

Life stage	Rv positive	*Bd* positive	Coinfected	Uninfected	Sample total (n)	Mean Rv load	Mean Rv SE	Mean *Bd* load	Mean *Bd* SE
Larva	0	0	0	3	**3**	-		-	
Paedomorph	3	1	2	5	**11**	34,796.7	±33,613.6	3,573.3	±3,329.3
Metamorphosing	3	1	2	0	**6**	3.07×10^7	$\pm 1.99 \times 10^7$	1.25×10^5	±95,949.8
Adult	5	7	2	6	**20**	3.86×10^7	$\pm 3.77 \times 10^7$	9,322.7	±56,66.4
Sample total (n)	**11**	**9**	**6**	**14**	**40**				

Supplementary Table 3. Summary of GLM and ANOVA test statistics of alpha diversity within and between *N. perstriatus* skin bacterial communities. Regression coefficients (β) from GLMs displayed with standard error. Significant effects (p < 0.05) are shown in bold.

Effect	Faith's Phylogenetic Diversity				ASV Richness				Shannon Diversity			
	F	β (±se)	t	p-value	F	β (±se)	t	p-value	F	β (±se)	t	p-value
GLM + ANOVA												
**Life stage**	**15.83**	**_**	**_**	**<0.00005**	**39.8**	**_**	**_**	**<0.00005**	**10.80**	**_**	**_**	**0.00009**
Infection status	0.59	_	_	0.21	0.91	_	_	0.45	1.11	_	_	0.36
Snout-vent length	0.66	_	_	0.66	1.01	_	_	0.32	0.46	_	_	0.50
Mass	0.56	_	_	0.16	2.78	_	_	0.11	0.00	_	_	0.97
Sampling date	0.87	_	_	0.51	1.14	_	_	0.37	0.28	_	_	0.94
GLM												
Intercept	_	8.81 (±5.00)	1.76	0.09	_	4.46 (±0.29)	15.5	<0.0001	_	2.68 (±0.30)	8.90	<0.00005
Paedomorph	_	0.34 (±5.60)	0.06	0.952	_	-0.05 (±0.32)	-0.15	0.88	_	-0.13 (±0.34)	-0.39	0.82
**Metamorphosing**	**_**	**13.24 (±6.13)**	**2.16**	**0.0376**	**_**	**1.13 (±0.35)**	**3.23**	**0.0027**	_	0.69 (±0.37)	1.86	0.074
**Adult**	**_**	**21.21 (±5.39)**	**3.93**	**0.00036**	**_**	**1.57 (±0.31)**	**5.09**	**0.000012**	**_**	**1.12 (±0.32)**	**3.47**	**0.00137**

Supplementary Table 4. Statistical results of pairwise PERMANOVA and PERMDISP models comparing skin bacterial communities in life stages of *N. perstriatus* for three metrics of beta diversity: Bray Curtis dissimilarity (BC), unweighted UniFrac distance (UW-UF), and weighted UniFrac distance (W-UF). Comparisons of life stages are denoted by pairs of letters, where L = larva, P = paedomorph, M = metamorphosing, and A = adult. Rows of pairwise comparisons containing significant values are bolded and asterisks denote level of significance (•p ~ 0.05, *p ≤ 0.05, **p ≤ 0.01, ***p ≤ 0.005).

	PERMANOVA						PERMDISP	
	Pair	Sum Of Squares	R^2	F	p-value	Adjusted p-value	Observed p-value	Permuted p-value
BC	**P-A**	**4.45**	**0.49**	**28.27**	**0.001*** **	**0.006** **	**<0.0001*** **	**0.001*** **
	**P-M**	**1.15**	**0.38**	**9.77**	**0.001*** **	**0.006** **	**0.001*** **	**0.002*** **
	**P-L**	**0.32**	**0.25**	**4.41**	**0.004*** **	**0.024***	0.45	0.46
	**A-M**	**0.77**	**0.13**	**3.44**	**0.001** **	**0.006** **	0.87	0.85
	**A-L**	**1.42**	**0.25**	**6.60**	**0.001*** **	**0.012** **	**0.051•**	**0.043***
	M-L	0.56	0.26	2.51	0.016	0.096	0.31	0.32
UW-UF	**P-A**	**1.67**	**0.20**	**7.10**	**0.001*** **	**0.006** **	0.23	0.22
	**P-M**	**0.67**	**0.15**	**2.87**	**0.002*** **	**0.012***	0.87	0.86 ·
	P-L	0.25	0.08	1.13	0.208	1.000	**0.004*** **	**0.008** **
	**A-M**	**0.39**	**0.07**	**1.63**	**0.011***	**0.066•**	0.41	0.40
	**A-L**	**0.76**	**0.14**	**3.24**	**0.001*** **	**0.006** **	**<0.0001*** **	**0.001*** **
	M-L	0.45	0.21	0.21	0.017	0.102	**0.005** **	**0.006** **
W-UF	**P-A**	**0.52**	**0.52**	**30.80**	**0.001*** **	**0.006 ** **	**0.0005*** **	**0.002*** **
	**P-M**	**0.12**	**0.41**	**11.34**	**0.001*** **	**0.006 ** **	0.06	0.07
	**P-L**	**0.05**	**0.32**	**5.99**	**0.005** **	**0.030***	0.62	0.64
	**A-M**	**0.09**	**0.15**	**4.15**	**0.001*** **	**0.006** **	0.40	0.37
	**A-L**	**0.15**	**0.25**	**6.63**	**0.002*** **	**0.012***	0.14	0.14
	M-L	0.04	0.23	2.14	0.075	0.450	0.54	0.55

Supplementary Table 5. Statistical results of pairwise PERMANOVA and PERMDISP models comparing skin bacterial communities between infection types in *N. perstriatus* for three metrics of beta diversity: Bray Curtis dissimilarity (BC), unweighted UniFrac distance (UW-UF), and weighted UniFrac distance (W-UF). Comparisons of infection types are denoted by pairs of letters, where *Bd* = *Batrachochytrium dendrobatidis*, Rv = Ranavirus, Co= Coinfection, and U = Uninfected. Rows of pairwise comparisons containing significant values are bolded and asterisks denote level of significance (•p ~ 0.05, *p ≤ 0.05, **p ≤ 0.01, ***p ≤ 0.005).

	PERMANOVA						PERMDISP	
	Pair	Sum Of Squares	R^2	F	p-value	Adjusted p-value	Observed p-value	Permuted p-value
BC	Bd-Co	0.31	0.08	1.18	0.26	1.00	0.69	0.98
	Bd-U	0.80	0.12	2.90	0.03*	0.19	0.44	0.80
	Bd-Rv	0.52	0.10	1.79	0.10	0.58	0.16	0.59
	Co-U	0.22	0.04	0.74	0.50	1.00	0.76	0.98
	Co-Rv	0.24	0.05	0.77	0.57	1.00	0.31	0.87
	Rv-U	0.25	0.03	0.79	0.51	1.00	0.64	0.96
UW-UF	Bd-Co	0.23	0.06	0.82	0.80	1.00	0.76	0.98
	Bd-U	0.42	0.06	1.50	0.09	0.522	0.51	0.87
	Bd-Rv	0.38	0.07	1.36	0.10	0.594	0.88	1.00
	Co-U	0.29	0.05	1.03	0.31	1.00	0.21	0.69
	Co-Rv	0.27	0.07	1.00	0.90	1.00	0.42	0.95
	Rv-U	0.28	0.04	0.99	0.49	1.00	0.48	0.93
W-UF	Bd-Co	0.02	0.06	0.82	0.49	1.00	0.89	1.00
	Bd-U	0.07	0.10	2.42	0.06•	0.34	0.21	0.52
	Bd-Rv	0.05	0.08	1.57	0.16	0.94	0.28	0.75
	Co-U	0.02	0.04	0.75	0.538	1.00	0.34	0.73
	Co-Rv	0.02	0.04	0.65	0.68	1.00	0.37	0.88
	Rv-U	0.02	0.02	0.51	0.837	1.00	0.75	0.99
